# Comparative volatile profiling of mango (*Mangifera indica*) and its role in mediating chemoecological behaviour of mango pulp weevil (*Sternochetus frigidus*)

**DOI:** 10.3389/finsc.2026.1724749

**Published:** 2026-02-04

**Authors:** Sayesta Mehejaveen Begum, Badal Bhattacharyya, Shimantini Borkataki, Bikash Hazarika, Dhanalakhi Gogoi, K Sindhura Bhairavi, Inee Gogoi

**Affiliations:** 1 Department of Entomology, College of Agriculture, Assam Agricultural University, Jorhat, India; 2 Department of Horticulture, College of Agriculture, Assam Agricultural University, Jorhat, India; 3 Department of Sericulture, College of Agriculture, Assam Agricultural University, Jorhat, India; 4 AICRP on Fruits, Assam Agricultural University, Jorhat, India

**Keywords:** chemoecology, GC-MS, hexane, mango, olfaction, *Sternochetus frigidus*, volatiles profiling, Y-tube olfactometer

## Abstract

**Introduction:**

The volatiles from Mango tree play a critical role in mediating interactions with herbivores, including the mango pulp weevil (*Sternochetus frigidus*), a serious quarantine pest of mango. The monophagous feeding habit and concealed life cycle of mango pulp weevil within fruits render insecticides, quarantine measures and post-harvest treatments largely ineffective. Identifying volatile cues emitted by the mango tree that mediate host location can aid in developing eco-friendly management strategies.

**Methods:**

In this study, Volatile Organic Compounds (VOCs) from different mango tree parts viz., young flush, mature flush, stem, flower, stone, whole and sliced green fruits, whole and sliced ripened fruits were characterized through solvent extraction and Gas Chromatography-Mass Spectrometry (GC-MS). The behavioural responses of adults were evaluated through Y-tube olfactometer assays.

**Results and discussion:**

Ripened mango fruits, young leaves and mango stone elicited the strongest responses and were further subjected to headspace volatile collection. Ripened fruit volatiles induced the highest levels of attraction (63.33%), activity (86.67%) and preference (46.15%). Young leaf volatiles induced moderate attraction (46.7%) with high activity (83.3% but low preference (12%). Mango stone volatiles, in contrast, evoked minimal activity (6.7%) and preference (10%). GC-MS profiling of headspace extracts revealed nine compounds each from ripened fruits and young leaves and seven from stones, with D-limonene, 3-carene and α-pinene dominating across all treatments. These findings highlight key VOCs associated with host selection by *Sternochetus frigidus* and provide a chemical basis for developing attractant-based trapping systems as components within a holistic Integrated Pest Management (IPM) approach in mango cultivation.

## 1 Introduction

Mango (*Mangifera indica* Linn.), a tropical fruit tree native to South Asia, is revered as the “King of Fruits”. It has gained widespread popularity and known to be consumed across diverse regions of the world. Mangoes stand as one of the most profitable fruit crops found in tropical and sub-tropical regions worldwide (1). It belongs to the family Anacardiaceae which includes generally woody plants, most of which produces edible fruits and nuts. The tree thrives best in warm, frost-free climates and tolerates a wide range of soils but is susceptible to hard freezes. This adaptable nature of mangoes enables farmers to cultivate them in any tropical region from sea level up to 1400 meters elevation. Mango orchards are common throughout India, including the North eastern states where the crop is grown on sub-tropical plains and foothills (2).

India is the largest producer of mangoes in the world, reporting approximately 56% of global production. The total area under mango cultivation in India is 2.41 million hectares (3), with a total production of 22.84 million tonnes (4). The main mango-producing states in India include Uttar Pradesh, Andhra Pradesh, Karnataka, Bihar, Gujarat and Maharashtra. Andhra Pradesh leads the mango production with 23.89% share and achieves the highest productivity (3^rd^ Advance Estimate, 2023, Department of Agriculture & Farmers Welfare, Ministry of Agriculture & Farmers Welfare, Government of India). India maintains its position as a leading exporter of fresh mangoes to international markets. The fresh mango exports from India reached 29938.38 metric ton (MT) during the year 2024-25 (https://agriexchange.apeda.gov.in/India/ExportSummery/Index) (5). The North eastern region of India, however contributes a minor share of national mango output as cultivation in these states is mostly in small land holdings (2).

Despite its economic significance, mango cultivation faces several challenges that threaten both yield and quality. Among these, pest infestations remain a major concern. Mango is attacked by a range of insect and non-insect pests (6), with insect pests posing the most serious threat to sustainable and profitable production. Key insect pests of economic importance include hoppers, fruit flies, fruit borers, pulp weevils and nut weevils. Moreover, the changing climatic conditions further exacerbate these issues by altering pest dynamics and infestation patterns, thereby seriously impacting mango production both directly and indirectly.

Among these insect pests, the mango pulp weevil (*Sternochetus frigidus*) is considered particularly destructive due to its designation as a major quarantine pest. This pest is highly host-specific, occurring exclusively on cultivated and wild mango species because of its strict monophagous feeding behaviour. Unlike many other pests that damage the external parts of the fruit, the grub of *S. frigidus* develops entirely within the fruit, feeding on the pulp and causing significant internal damage that often goes unnoticed until harvest, making it a serious threat to both domestic consumption and export quality standards. Detection of the damage becomes difficult because infested fruits often appear externally healthy and the infestation becomes obvious when the adults bore exit holes. Adult weevils start their life cycle by emerging from stones then move upward through fruit pulp until they create scars that allow microbial infections to begin which makes the fruit unusable by humans (7). The problem intensifies because the different pests simultaneously harm the fruiting structures while consuming the fruit and moving quickly and producing many offspring and multiple life cycles within a short period. The life cycle of *S. frigidus* perfectly matches the mango fruiting season. The female weevil places each egg individually on the fruit surface then covers it with protective substance which turns into a protective plug that holds the eggs in position (8). The eggs develop inside the fruit pulp for nine days before hatching into five larval stages that complete their development within 20 days while creating tunnel structures (9). The mature larvae create a pupal chamber inside the fruit before pupation occurs and adults escape through visible exit holes that appear after fruit decay or drop (9).

The total damage from these pests reaches millions of dollars in various important economic species. Food security demands immediate research enhancement to protect crop harvests against insect pests. The implementation of pesticides leads to higher costs and environmental damage as well as potential negative impacts on consumer and producer health. The insidious pest would be effectively managed through the exploitation of volatile extracts derived from plant parts using ecofriendly approaches. Despite being native to Assam, comprehensive chemoecological studies on *S. frigidus* host-plant interactions are lacking. Hence, research dedicated to the host volatile behavioural responses of *S. frigidus* is essential because of the economic value of mango and the quarantine significance of the pest species. The study of chemical ecology between these interactions will lead to the development of environmentally safe pest management approaches that minimize post-harvest losses while maintaining mango fruit quality and export potential. The present investigation adopted an integrated chemoecological approach to examine host mediated interactions between mango and *S. frigidus* by combining dynamic headspace volatile collection, Gas Chromatography-Mass Spectrometry characterisation and behavioural validation through olfactometer bioassays. While earlier studies on mango volatiles or host association of *S. frigidus* have largely remained descriptive, the current work advances existing knowledge by directly linking identified volatile constituents with experimentally validated olfactory responses. In addition, the comparative assessment of volatiles derived from different mango tissues provides new insight into host selection cues operating at the adult stage and establishes a scientific foundation for the development of semiochemical based, environmentally compatible management strategies.

## 2 Materials and methods

### 2.1 Test fruit

The mango variety selected for this investigation was a local popular cultivar commonly cultivated in Assam. This variety was chosen based on its availability, documented susceptibility to *S. frigidus* and its significance in regional mango cultivation practices.

### 2.2 Insects

Adult weevils were collected during the same time period from infested mango fruits in and around the Assam Agricultural University campus including the Horticulture Experimental Farm and Animal Husbandry and Dairying Farm, Assam Agricultural University, Jorhat. The collected insects were subsequently maintained under laboratory conditions to establish a uniform experimental population and were further employed for all bioassays to ensure consistency in age and physiological status.

### 2.3 Design and layout

The experiment was designed following a Completely Randomized Design (CRD) comprising nine treatments *viz.*, young mango leaves, old mango leaves, young petioles, stem, flowers, young mango fruit, ripened mango fruit, mango bark and mango stone with three replications each. For each treatment, thirty (30) insects preferably of uniform developmental stage were released into the Y-tube olfactometer with no sex-based differentiation.

### 2.4 Preparation of host plant extract

Host plant extracts were prepared using freshly collected mango plant parts which were further used as treatments. For each treatment, 50 g of the specific plant material was soaked in 100 ml of HPLC-grade hexane in clean glass beakers. The plant material was left to macerate at room temperature for 24 hours to allow extraction of volatile compounds. The supernatant from each sample was carefully separated and transferred to fresh beakers after soaking. These extracts were then concentrated by placing them in a water bath at 60°C until the volume was reduced to approximately 1 ml. The concentrated extracts were refrigerated until further use in olfactometer bioassays.

### 2.5 Y-tube olfactometer bioassays

To evaluate the olfactory response of *Sternochetus frigidus* adults to different mango plant volatiles, a Y-tube olfactometer was used. The apparatus consisted of a central stem (15 cm long) and two arms (each 7.5 cm) set at an angle of 60° with an internal diameter of 2.3 cm. Each arm was connected to an odour chamber, one holding the test odour and the other the control. Purified air was drawn through the olfactometer using a Syntech air-delivery system maintaining a steady flow of 250 ml/min in each arm. For each test 1µL of the volatile extract was applied to a 1 × 2 cm strip of Whatman filter paper and placed at the end of one arm while a control strip treated with the same volume of HPLC-grade hexane was placed in the opposite arm. Individual adult weevils were released at the entrance of the central stem and allowed up to 10 minutes to make a choice between the test odour and control. Filter papers were changed after every three insects to maintain consistency and avoid cross-contamination. A total of 30 adults were tested per treatment per replication. After each replicate, the Y-tube apparatus was thoroughly cleaned, first with acetone and then air-dried in an oven to eliminate any residual volatiles. Each weevil was used only once and then discarded from further trials.

### 2.6 Behavioural observations

Behavioural responses of adult *Sternochetus frigidus* were assessed using a Y-tube olfactometer under controlled laboratory conditions, with each insect observed for a duration of ten minutes. Movement was recorded as the total number of complete entries into each arm of the olfactometer while activity encompassed all locomotory behaviours including walking, turning, probing and antennal scanning. Preference was determined as the proportion of insects selecting the treatment arm, with the first choice considered indicative of olfactory attraction. Attraction was inferred when a significant proportion of insects consistently chose and spent more time in the treatment arm relative to the control while repulsion was concluded when insects predominantly avoided the treatment arm. All bioassays were conducted under uniform environmental conditions to prevent interference providing a consistent and reproducible framework for evaluating insect behavioural responses.

### 2.7 Headspace sampling

**Figure 1:**
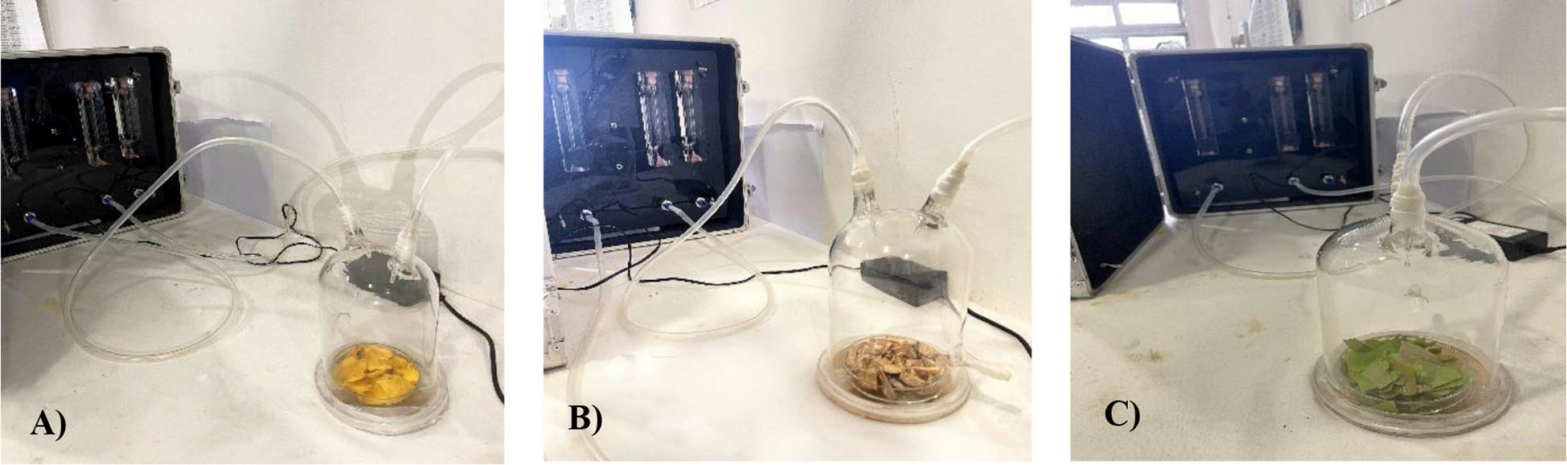
Headspace volatile collection. **(A)** Ripened mango fruit. **(B)** Mango stone. **(C)** Young mango leaves.

Three most effective treatments were selected and freshly collected for headspace volatile collection using Porapak-Q (80–100 mesh) filters for 6 hours and was eluted with n-hexane and re-evaluated for confirmatory bioassays (**Figure 1**). For volatile collection, Porapak-Q was packed into 8 cm glass tubes of 2.5 mm internal diameter. These tubes were connected to a dynamic headspace system driven by a flowmeter which delivered a constant airflow of 20 mL per minute for six hours. Prior to exposure of the test materials, Porapak-Q filters were pre eluted with n-hexane and subjected to identical airflow conditions in the absence of plant material. The eluates obtained from these runs were designated as blank controls to account for background contaminants originating from the adsorbent, solvent and sampling apparatus. The plant samples were then confined in 2 litre glass entrainment chambers and sealed with paraffin wax to ensure airtight closure. The trapped compounds were eluted with n-hexane, left for 10–15 minutes to ensure full extraction and subsequently transferred into clean vials for storage at -20 °C until testing for later bioassay.

### 2.8 Identification of plant volatiles

The same host plant extracts employed for the olfactometer and headspace bioassays in the experiment were subjected to Gas Chromatography-Mass Spectrometry (GC-MS) analysis, thereby ensuring exact correspondence between the chemical profiles and the observed olfactory responses and to detect and identify th specific volatile compounds present in them. The GC-MS analysis was carried out in the Chemical Ecology Laboratory, Department of Entomology, AAU, Jorhat. A ThermoFisher ISQ 7000 unit was employed operating with a TG-5MS column (30 m × 0.25 mm × 0.25 μm) and helium as the carrier gas under a constant flow of 1 ml per minute. The thermal programme raised the temperature from 40°C to 250°C in stages which enabled full chromatographic separation of the volatile extracts resulting in a total run time of 25 minutes. Ionisation was performed at 70 eV with both the ion source and transfer line held at 250°C. Peak identification was achieved through spectral comparison against the NIST11.L reference library.

### 2.9 Statistical analysis

Behavioural responses were analysed using both parametric and non-parametric methods combining ANOVA with Tukey’s HSD, Chi-square, Mann-Whitney U, Kruskal-Wallis with Dunn’s test, Rank-Biserial correlation and Critical Difference.

## 3 Results

### 3.1 Behaviour

The behavioural responses of *S. frigidus* from the initial olfactometer bioassays demonstrated considerable heterogeneity across the nine evaluated treatments. Results expressed in Mean ± SD of 30 samples from each set followed by Tukey’s value in the same criterion that are significantly different from each other by Tukey’s HSD Test at probability 0.05 (**Table 1**). The statistical analysis revealed significant variations amongst treatments (p<0.01), with ripened mango fruit demonstrating maximum attractancy (15.33 ± 0.58) whilst young petioles exhibited maximum repellent properties (14.67 ± 0.58). The analysis also revealed highly significant preferences (p<0.01) for ripened mango fruit (χ²=9.328) followed by young mango leaves (χ²=5.882) and mango stone (χ²=2.722). In contrast, the other six treatments demonstrated statistically significant yet comparatively moderate levels of attraction (p<0.05) (**Table 2**).

**Table 1:** Variations in the responses of *Sternochetus frigidus* to volatiles associated with vegetative, flowering and fruiting stages of mango in Y-tube Olfactometer bioassay.

Treatment	Attraction	Repulsion	Neutral
T_1_	14.67 ± 1.15^bc^	8.00 ± 1.00^a^	7.33 ± 1.15^ab^
T_2_	11.67 ± 1.15^ab^	8.67 ± 2.89^ab^	9.67 ± 2.52^bc^
T_3_	11.33 ± 1.53^ab^	14.67 ± 0.58^c^	4.00 ± 1.73^a^
T_4_	11.00 ± 1.00^a^	13.00 ± 2.65^bc^	6.00 ± 2.65^ab^
T_5_	12.00 ± 1.00^abc^	5.33 ± 1.15^a^	12.67 ± 2.08^c^
T_6_	12.00 ± 1.00^abc^	8.67 ± 2.08^ab^	9.33 ± 1.53^bc^
T_7_	15.33 ± 0.58^c^	7.00 ± 1.00^a^	7.67 ± 1.15^abc^
T_8_	14.00 ± 1.00^abc^	7.67 ± 0.58^a^	8.33 ± 1.53^abc^
T_9_	14.33 ± 2.08^abc^	9.67 ± 0.58^ab^	6.00 ± 1.73^ab^
F value	5.311	10.080	5.540
p-value (≤0.05)	0.002*	0.001*	0.001*

Results expressed in mean ± SD of 30 samples. Means within the same column followed by different lowercase letters are significantly different according to Tukey’s HSD test at *p* ≤ 0.05.

*Significant at 0.05.

**Table 2:** Response of *Sternochetus frigidus* to volatiles associated with vegetative, flowering and fruiting stages of mango in Y-tube Olfactometer bioassay.

Treatment	Attraction	Repellence	χ²	p
Young mango leaves	44	24	5.882*	0.015 (p<0.05)
Old mango leaves	35	26	1.328	0.249 (p<0.05)
Young petioles	34	44	1.282	0.257 (p<0.05)
Stem	33	39	0.500	0.480 (p<0.05)
Flowers	36	16	7.692**	0.005 (p<0.01)
Young mango fruit	36	26	1.613	0.204 (p<0.05)
Ripened mango fruit	46	21	9.328**	0.002 (p<0.01)
Mango bark	42	23	5.554	0.018 (p<0.05)
Mango stone	43	29	2.722*	0.029 (p<0.05)

*Significant at 0.05, **Significant at 0.01.

The comprehensive analysis of weevil behavioural activity and preferences revealed substantial treatment-specific variations (**Table 3**). Young petioles (T_3_) elicited highest activity levels (86.67%) while simultaneously demonstrating negative preference (-13.05%) implying elevated exploratory activity aligned with avoidance driven responses. Conversely, notable positive activity as well as preference values were recorded for ripened mango fruit (T_7_) followed by young mango leaves (T_1_) and mango stone (T_9_).

**Table 3:** Variations in per cent activity and preference of *Sternochetus frigidus* towards treatments during Y-tube Olfactometer bioassay.

Treatment	Activity (%)	Preference (%)
T_1_	75.56^ab^	29.29^ab^
T_2_	67.78^b^	15.96^bc^
T_3_	86.67^a^	-13.05^c^
T_4_	80.00^ab^	-7.75^c^
T_5_	57.78^c^	38.95^a^
T_6_	68.89^b^	16.71^bc^
T_7_	74.44^ab^	35.89^a^
T_8_	72.22^ab^	29.24^ab^
T_9_	76.67^ab^	19.03^bc^
H statistic	15.76	17.05
*p-*value (*p* < 0.05)	0.04	0.02

*Significant at 0.05, **Significant at 0.01.

Values sharing the same lowercase letter within a column do not differ significantly, whereas values followed by different letters indicate significant differences at *p* ≤ 0.05.

### 3.2 Selection and validation of best treatments

Based upon the preliminary screening results, ripened mango fruit, young mango leaves and mango stone were identified as the most potent treatments out of all nine treatments and were further subjected for comprehensive headspace volatile collection and confirmatory bioassays. The behavioural inclination of *S. frigidus* amongst the three most efficacious treatments was statistically scrutinised using chi-square tests to determine the prominence of attraction over repulsion. Analysis indicated that among the top three treatments, ripened mango fruit evoked a statistically significant preference (5.538, *p* < 0.05) with 19 instances of attraction against 7 of repulsion in *S. frigidus*. In comparison, responses to young mango leaves and mango stone yielded non-significant behavioural responses implying no clear preference (**Table 4**).

**Table 4:** Response of *Sternochetus frigidus* to volatiles associated with best treatments during Y-tube Olfactometer bioassay.

Treatment	Attraction	Repellency	χ²	*p*
Ripened mango fruit	19	7	5.538*	0.018 (*p* < 0.05)
Young mango leaves	14	11	0.360	0.548 (*p* < 0.05)
Mango stone	11	9	0.200	0.654 (*p* < 0.05)

*Significant at 0.05.

In order to validate whether the three most promising volatile treatments produced significantly heterogeneous behavioural infestations, a comprehensive array of non-parametric inferential tests were undertaken (**Table 5**). Despite subtle differences evident across individual pairwise comparisons, perusal of data presented in **Table 5** demonstrated that the behavioural responses of *S. frigidus* to the best three volatile treatments did not differ significantly with respect to attraction, repellence and neutrality remaining statistically comparable, indicating uniform chemotactic behaviour within the parameters of the experimental setup. The comparative analysis illustrated varying responses of the insect towards the test volatiles, thereby validating the ranking of their attractive potential. Adult *S. frigidus* elicited significantly heightened chemotactic engagement to volatiles of ripened mango fruit marked by 63.33% directed movement, 86.67% overall activity and a peak preference index of 46.15%. Young mango leaves also generated notable movement and activity of 46.67% and 83.33% respectively, yet the preference remained low (12%) (**Table 3**). Mango stone elicited the weakest response with limited movement (36.67%), low activity (6.67%) and minimal preference (10%) (**Table 6**).

**Table 5:** Variations in the responses of *Sternochetus frigidus* to best treatments during Y-tube Olfactometer bioassay.

Comparison	Test Type	Test Statistic	Critical Value (α = 0.05)	p-value
T_1_ vs T_2_	Mann-Whitney Test (U)	2.00	U_a_ = 2.00	p < 0.05
	Dunn’s *Post Hoc* (Z)	-0.52	Z_a_ = 1.96	p > 0.05
T_1_ vs T_3_	Mann-Whitney Test (U)	2.00	U_a_ = 2.00	p < 0.05
	Dunn’s *Post Hoc* (Z)	-0.38	Z_a_ = 1.96	p < 0.05
T_2_ vs T_3_	Mann-Whitney Test (U)	1.50	U_a_ = 2.00*	p < 0.05
	Dunn’s *Post Hoc* (Z)	0.15	Z_a_ = 1.96	p < 0.001
All 3 groups	Kruskal-Wallis Test (H)	0.29	χ²_a_ (df = 2) = 5.99	p < 0.001
All 3 groups	Chi-square Test (χ²)	6.37	9.48	p < 0.001

*Significant at 0.05.

Different lowercase letters indicate statistically significant differences among treatments at *p* ≤ 0.05 based on non-parametric multiple comparison test.

**Table 6:** Variations in per cent movement, activity and preference of *Sternochetus frigidus* towards best treatments in Y-tube Olfactometer bioassay.

#	Movement (%)		Activity (%)	Preference (%)
	Treatment	Control		
Ripened mango fruit	63.33	23.33	86.67	46.15
Young mango leaves	46.67	36.67	83.33	12.00
Mango stone	36.67	30.00	6.67	10.00

### 3.3 Gas chromatography-mass spectrometry profiling of volatile compounds from the three most potent treatments

**Figure 2:**
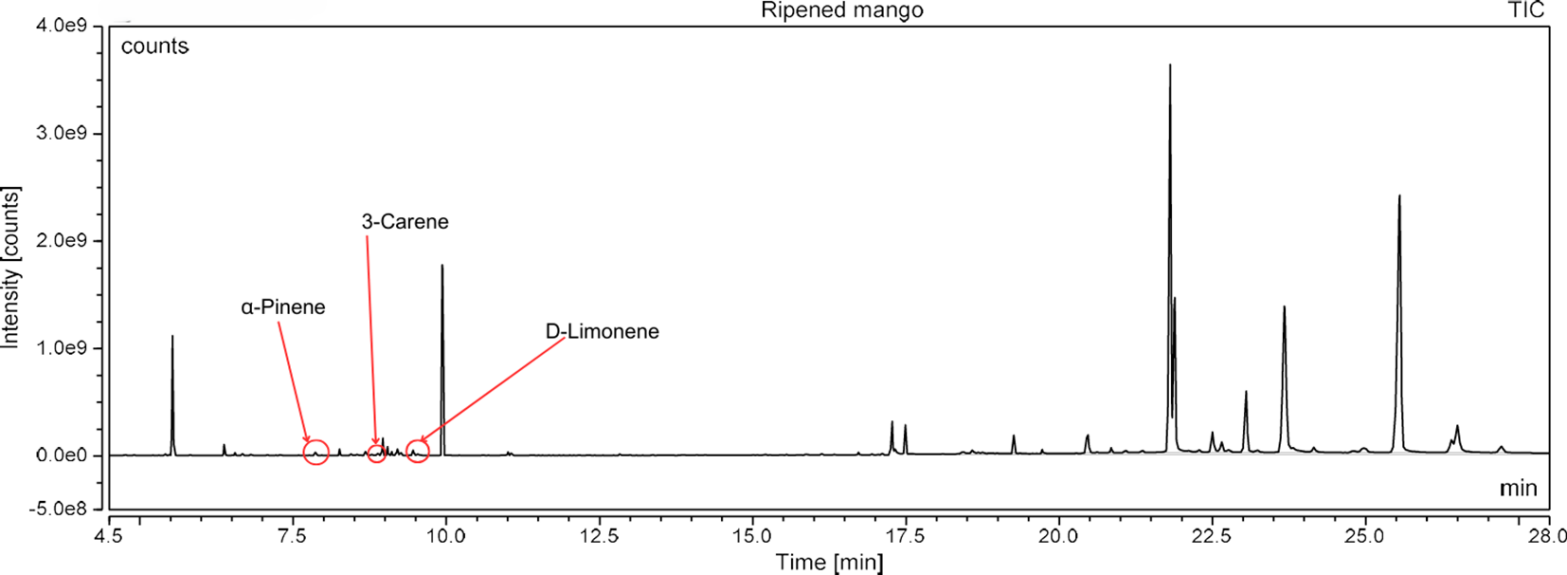
GC-MS analysis of ripened mango fruit.

**Figure 3:**
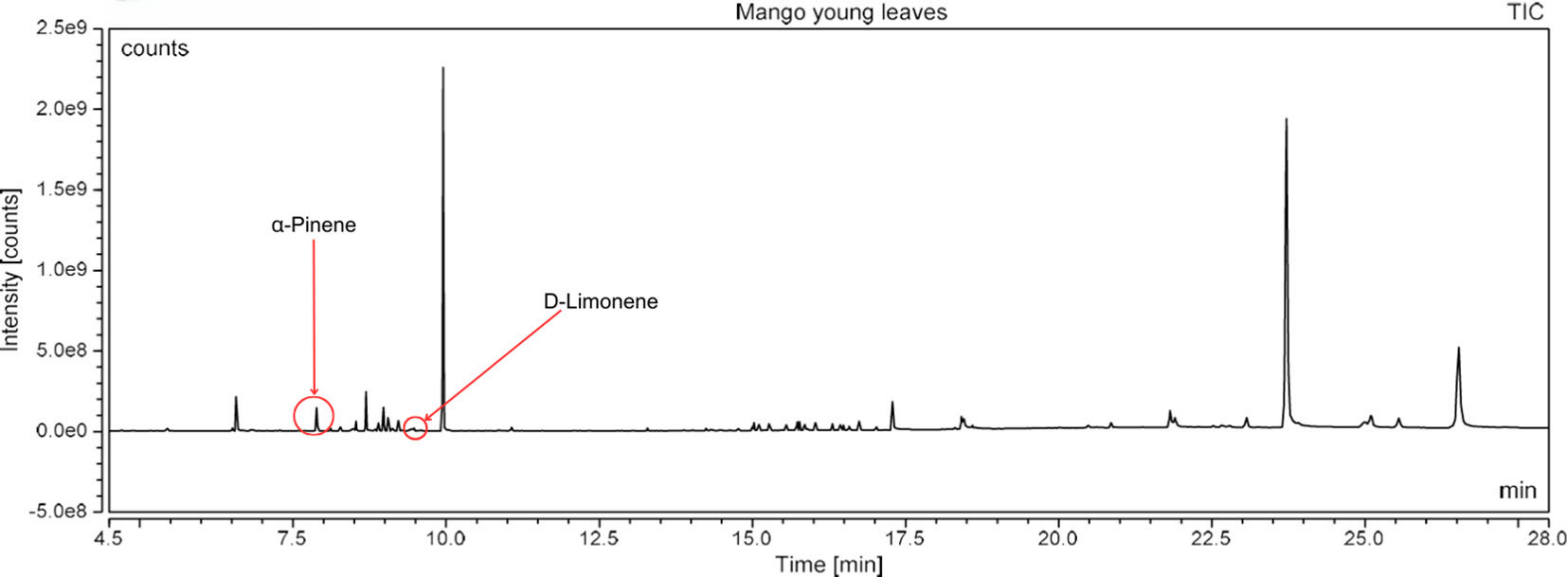
GC-MS analysis of young mango leaves.

**Figure 4:**
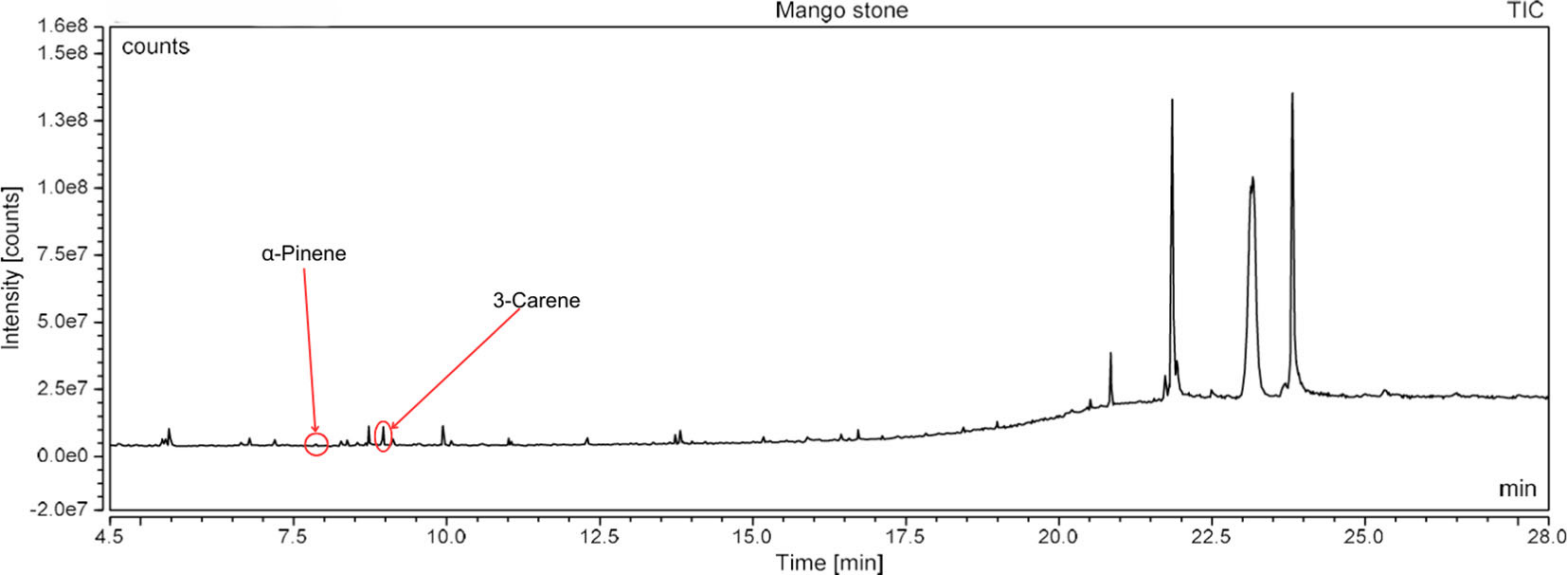
GC-MS analysis of young mango stone.

Following a thorough sequence of behavioural bioassays on nine volatile substrates derived from mango, Gas Chromatography-Mass Spectrometry (GC-MS) was performed to decipher the volatile constituents responsible for triggering olfactory responses in *S. frigidus* (**Figures 2**–**4**). On the basis of inferential statistical validation and preferences derived from Y-tube olfactometer bioassays, ripened mango fruit, young mango leaves and mango stone were identified as the three most potent treatments in eliciting behavioural attraction. Consequently, volatiles from these best treatments were extracted through headspace and again subjected to detailed GC-MS profiling to identify pivotal compounds responsible in mediating host-recognition cues. A total of 25 compounds were found across these treatments, 9 from ripened mango fruit and young mango leaves each and 7 from mango stone, which included a combination of monoterpenes, esters, hydrocarbons, aldehydes, alcohols and other chemically significant volatiles. Among these identified compounds, D-Limonene, 3-Carene and α-Pinene were consistently present in all treatments indicating their prevalence within the headspace blend. , **Table 7** provides an overview of all the compounds that were identified across the potent treatments along with their source substrates and respective functional role in inducing host-plant interactions.

**Table 7:** Major volatile compounds detected in the three best mango substrates viz., ripened mango fruit (T_1_), young mango leaves (T_2_) and mango stone (T_3_) and their functional roles.

Sl. No.	Compound	Retention time (min)	Structure	Treatment	Importance
1.	D-Limonene	T_1_: 9.201 T_2_: 9.218	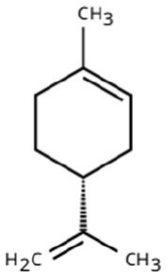	Ripened Mango Fruit & Young Mango Leaf	Monoterpene with fruity, citrus aroma; attracts fruit-feeding beetles and weevils; facilitates host-seeking and feeding behaviour.
2.	3-Carene	T_1_: 8.963 T_3_: 8.963	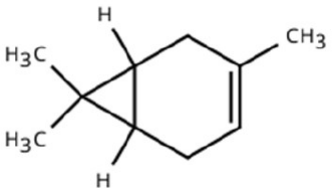	Ripened Mango Fruit & Mango Stone	Sweet, pungent aroma; mediates host recognition and oviposition cues for coleopterans and bark beetles.
3.	α-Pinene	T_1_: 7.878 T_2_: 7.888 T_3_: 7.868	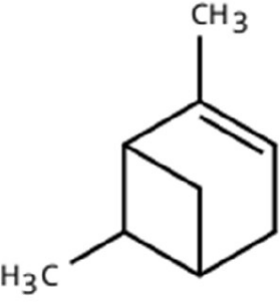	All (Fruit, Leaf & Stone)	Pine-like volatile; potent olfactory attractant; induces landing and host-seeking behaviour in bark beetles and weevils.
4.	Ethyl butanoate	T_1_: 5.531	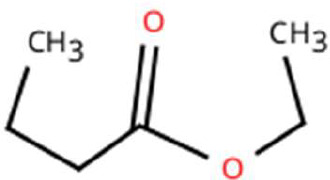	Ripened Mango Fruit	Imparts sweet, fruity odour; known to attract several frugivorous insects
5.	Ethyl tiglate	T_1_: 7.956	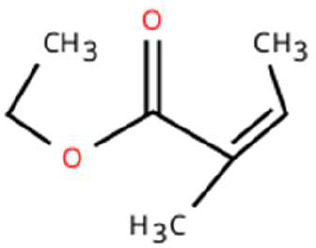	Ripened Mango Fruit	Fruity-sweet volatile; mimics signals of fruit maturity.
6.	Hexadecanoic acid	T_1_: 17.275 T_2_: 17.285	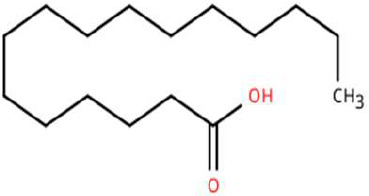	Ripened Mango Fruit & Young Mango Leaf	Fatty acid involved in wax biosynthesison leaves; provides physical barrier and antimicrobial protection.
7.	3-Hexen-1-ol, (Z)-	T_2_: 6.572	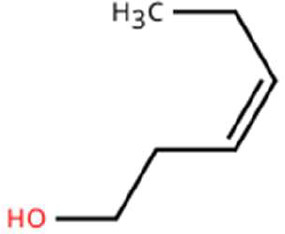	Young Mango Leaf	Known as a green leaf volatile (GLV); released upon leaf damage, signals herbivory and may attract phytophagous insects and herbivores
8.	Caryophyllene	T_1_: 13.374 T_2_: 13.388	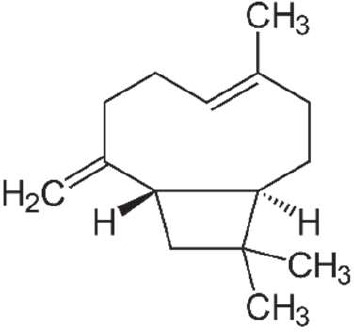	Ripened Mango Fruit & Young Mango Leaf	Sesquiterpene, known attractant or modulator for certain coleopterans; may enhance attractiveness in combination with other volatiles
9.	Heptadecane	T_1_: 14.677 T_2_: 14.690	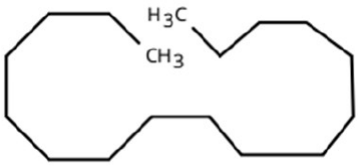	Ripened Mango Fruit & Young Mango Leaf	Long-chain alkane; contributes to host surface cues and tactile recognition during insect landing.
10.	Benzene derivatives	–	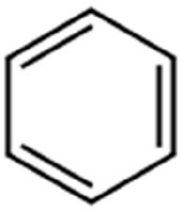	Ripened Mango Fruit	Components of mango volatiles; act as attractants in blend.
11.	β-Myrcene	T_2_: 8.694	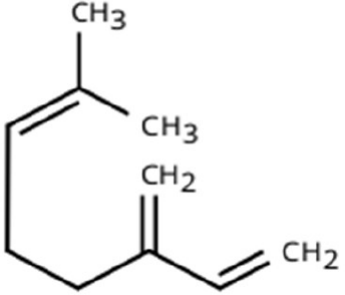	Young Mango Leaf	Monoterpene with sweet, resinous odour; involved in plant-insect interactions; contributes to fruit aroma and may act as location cues of host for weevils.
12.	Octadecane and derivatives	–	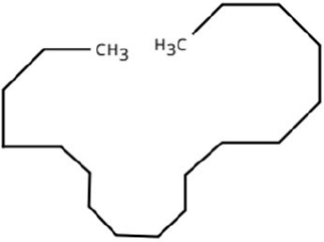	Young Mango Leaf	Hydrocarbons contributing to cuticular wax, influencing leaf surface characteristics and insect adherence.
13.	Tridecane	T_2_: 12.102	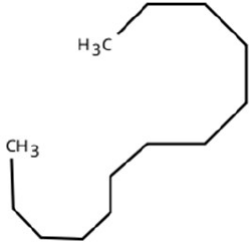	Young Mango Leaf	Alkane contributing to surface texture; influences orientation and probing behaviour.
14.	Cyclohexene derivatives	–	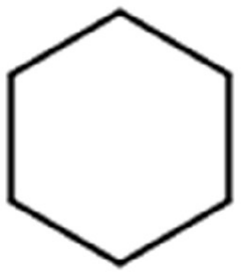	Young Mango Leaf	Cyclic hydrocarbonknown to influence insect movement or antennal responses in Y-olfactometer tests
15.	Cyclohexane	T_1_: 2.854 T_2_: 2.725 T_3_: 2.616	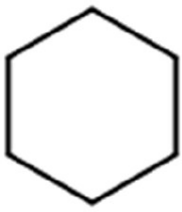	All (Fruit, Leaf & Stone)	Volatile hydrocarbon; acts as an olfactory stimulant for herbivores.
16.	Heptane	T_1_: 3.330 T_2_: 3.225 T_3_: 3.143	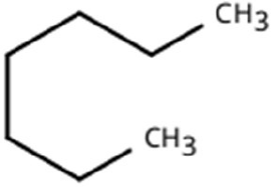	All (Fruit, Leaf & Stone)	Alkane; may mimic host plant volatiles.
17.	Cyclohexane, methyl-	T_1_: 3.769 T_2_: 3.674 T_3_: 3.603	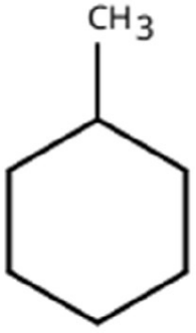	All (Fruit, Leaf & Stone)	Methylated cycloalkanecontributes to host aroma and may act as location cues of host
18.	Phenylethyl alcohol	T_3_: 10.228	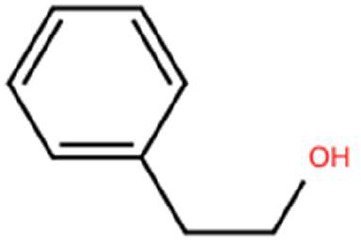	Mango Stone	Aromatic alcohol; contribute to floral aroma; may act as attractant for phytophagous herbivores including coleopterans
19.	Phenol, 3-(10Z)-10-nonadecen-1-yl-	T_3_: 23.816	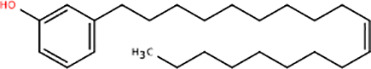	Mango Stone	Long-chain phenolic compound; high abundance; acts as semiochemical.

‘-’ refers to multiple retention time representing mixture of compounds.

## 4 Discussion

### 4.1 Behavioural response to volatiles

The Y-tube Olfactometer bioassays conducted during this study revealed clear preferences of *S. frigidus* adults for specific mango plant volatiles. In preliminary screening of nine volatile extracts, ripened mango fruit consistently elicited the strongest attraction followed by young green mango leaf and mango stone volatiles. These results suggested that adult weevils were most responsive to fruit pulp cues consistent with their ecological niche as pulp feeders. This study generally supported earlier work on olfactory behaviour of mango pulp weevil. De Jesus (10) reported notable attraction of both sexes of *S. frigidus* towards the volatiles from green mango fruit and male frass with virgin females showing 73.30% attraction to frass and 63.30% attraction to green fruit volatiles. The stage-dependent volatile shifts in our ripened fruit samples align with established fruit-maturation physiology documented in citrus (11) and lemon (12), where monoterpene concentrations increase progressively during ripening. Expanding on these observations, the present study suggested that ripe mango fruits might have emitted key chemical cues effective in eliciting attraction due to presence of similar chemical compounds such as acetic acid and decane previously identified by De Jesus et al. (9) in mango flowers but their detection in our ripened fruit samples alongside elevated D-limonene and 3-carene reflects a distinct ripeness-associated volatile profile.

The relatively weaker response to leaf volatiles likely reflected the biological behaviour of *S.* frigidus. Although adults were known to feed on foliage during the mating season (13), volatiles from foliage seemed to act merely as general host cues. As Bruce et al. (14) asserted that phytophagous insects often respond to specific blends rather than single volatiles, the blend emitted by young leaves in the present study did induce movement but failed to provoke a definitive directional response suggesting a general arousal effect than precise orientation. No innate repulsion was observed for any mango volatile, aligning with the findings of Dudareva et al. (15) who proposed that such cues typically facilitated host recognition rather than deterrence. Statistical analysis confirmed a highly significant preference for fruit volatiles over leaf and seed. Similar olfactometer studies in other weevil species have shown that host-specific volatile blends heavily influenced behavioural choices (16).

To validate the preliminary results, headspace volatiles were collected and re-evaluated from the three substrates that had shown greatest attractiveness *viz.*, ripened mango fruit, young mango leaf and stone. The confirmatory bioassays essentially produced the same preference pattern in which ripened mango fruit volatiles drew highest attraction of 63.33% followed by young mango leaf volatiles with intermediate response of 46.67% and stone volatiles with minimal attraction of 36.67%. Although the attractiveness to mango stone volatiles placed them among the best three treatments in the present study, the findings did not align with earlier assumptions made by Monawarahmad (17) who clearly distinguished *S. frigidus* as a pulp feeder, unlike *S. mangiferae*, which was capable of feeding on the seed. It is plausible that the specific volatile compounds emitted by the mango stone either mimicked those associated with the pulp or triggered exploratory behaviours unrelated to direct feeding.

To sum up, the Y-tube olfactometer bioassays demonstrated that ripened mango fruit volatiles were the dominant olfactory attractant for *S. frigidus*. This observation concurred with general entomological principles (14) and the earlier research of De Jesus (10) though there was a shift in focus to a more advanced fruit stage likely because of the methodological differences particularly in the selection of volatile sources.

### 4.2 GC-MS profiling of volatiles and identification of active compounds

Chemical profiling using Gas Chromatography-Mass Spectrometry (GC-MS) of the headspace volatiles from ripened mango fruit, young mango leaf and mango stone supported the earlier behavioural data by characterising their volatile compositions. Nine volatile compounds were detected in both fruit and leaf samples whereas the seed volatiles yielded only seven. Each volatile profile was dominated by monoterpenes, particularly D-limonene, 3-carene and α-pinene. Other volatiles such as ethyl butanoate, ethyl tiglate, caryophyllene, (Z)-3-hexenol and phenylethyl alcohol were also detected in fruit and leaf samples. These findings aligned well with previous studies on mango volatiles where Pino et al. (18) reported D-limonene and 3-carene as dominant monoterpenes across multiple mango cultivars while the present study employing dynamic headspace collection from ripened fruit confirms that these compounds are prominent constituents of odour blends associated with increased attraction of *S. frigidus* at the whole-odour level without assigning effects to single compounds. Importantly, the key volatiles identified in this study *viz.*, D-limonene, 3-carene and α-pinene have documented ecological roles in other insect-plant systems. D-limonene has been identified as a potent male attractant in adult fruit fly *Bactrocera minax* by Cheng et al. (19), while β-myrcene elicited strong olfactory responses in adult fruit fly *B. dorsalis* as reported by Jayanthi et al. (20), both implicating these monoterpenes in host recognition in tephritid flies rather than specifically in *S. frigidus*. The findings of Jaleel et al. (21) revealed that female *Bactrocera dorsalis* and *B. correcta* exhibited pronounced olfactory responses to 3-carene under both laboratory and field conditions, suggesting a conserved ecological role for this monoterpene across diverse frugivorous insects even though its role in *S. frigidus* remains to be tested directly. In a similar context, α-pinene has been documented as a significant olfactory cue in mediating insect behaviour in a study conducted by Gerofotis et al. (22) on olive fruit flies, *Bactrocera oleae*, where exposure to its aroma markedly enhanced mating performance in both males and females. Taken together, these external studies indicate that several of the compounds detected here can function as semiochemicals in other taxa but in the present work they should be regarded as candidate cues rather than confirmed effectors for *S. frigidus* because compound-specific bioassays and electrophysiological recordings were not performed. In summary, the volatile composition corresponded closely with the observed behavioural responses at the level of intact odour sources. Ripened mango fruit which induced the strongest attraction was characterised by comparatively higher concentrations of D limonene, 3 carene and ester compounds than those detected in leaf or seed tissues. Prior research has established the relevance of such monoterpenes in host selection across insect taxa, which renders them plausible candidates for further targeted study in *S. frigidus* using electrophysiology and single-compound or blend bioassays.

## 5 Conclusions

Behavioural bioassays revealed a clear preference for volatiles from ripened fruit indicating a specialised preference for mature pulp, particularly for feeding. The identification of key volatiles with attractive and repellent properties presented significant potential for applied pest management. Synthetic lures modelled on volatiles derived from pulp may be developed for use in trap-based surveillance. These approaches hold promise in reducing chemical pesticide reliance and fostering environmentally responsible pest management. This research, by bridging behavioural bioassays and volatile profiling proposes a holistic and scientifically grounded strategy for managing *S. frigidus* populations in mango systems.

## Data Availability

The original contributions presented in the study are included in the article/supplementary material. Further inquiries can be directed to the corresponding authors.
